# Computer-Aided Screening and Revealing Action Mechanism of Food-Derived Tripeptides Intervention in Acute Colitis

**DOI:** 10.3390/ijms232113471

**Published:** 2022-11-03

**Authors:** Huifang Ge, Ting Zhang, Yuanhu Tang, Yan Zhang, Yue Yu, Fangbing Men, Jingbo Liu, Yiding Yu

**Affiliations:** 1 Jilin Provincial Key Laboratory of Nutrition and Functional Food, College of Food Science and Engineering, Jilin University, Changchun 130062, China; 2 State Key Laboratory of Tea Plant Biology and Utilization, School of Tea and Food Science and Technology, Anhui Agricultural University, Hefei 230036, China

**Keywords:** food-derived tripeptides, acute colitis, computer-aided screening, network pharmacology and bioinformatics, molecular docking

## Abstract

Food-derived tripeptides can relieve colitis symptoms; however, their alleviation mode has not been systematically evaluated as an alternative nutritional compound. This study aimed to reveal the potential mechanism of 8000 food-derived tripeptides against acute colitis using a computer-aided screening strategy. Forty-one potential hub targets related to colitis with a Fit score > 4.0 were screened to construct the protein-protein and protein-tripeptide network based on the PharmMapper database and STRING software (Ver. 11.5). In addition, 30 significant KEGG signaling pathways with *p*-values < 0.001 that the 41 hub targets mainly participated in were identified using DAVID software (Ver. 6.8), including inflammatory, immunomodulatory, and cell proliferation and differentiation-related signaling pathways, particularly in the Ras- and PI3K-Akt signaling pathways. Furthermore, molecular docking was performed using the Autodock against majorly targeted proteins (AKT1, EGFR, and MMP9) with the selected 52 tripeptides. The interaction model between tripeptides and targets was mainly hydrogen-bonding and hydrophobic interactions, and most of the binding energy of the tripeptide target was less than −7.13 kcal/mol. This work can provide valuable insight for exploring food-derived tripeptide mechanisms and therapeutic indications.

## 1. Introduction

Inflammatory bowel disease (IBD) has been recognized as a chronic, incurable, and idiopathic disease spreading worldwide. Therefore, alleviating IBD has become an emerging challenge for public health [1,2,3]. Acute colitis is a common intestinal disease; it will develop into IBD without reasonable intervention. Factors, such as oxidative stress, dysregulated immune responses, and nutrition deficiency, have been reported to be negatively related to colon health, which can aggravate colitis symptoms [4,5,6]. Currently, methods of relieving colitis are mainly based on drug intervention, commonly including aminosalicylic acid preparations, steroids, and glucocorticoids [6]; however, the strong side effects severely restrict their more comprehensive application. Thus, an alternative method for alleviating colitis that is efficient and safe is urgently needed.

Small-molecule active peptides extracted from food-derived proteins have nutritional supplementation [5] and physiological regulation functions, such as antioxidant, anti-inflammatory, and antibacterial functions [7,8,9,10,11]. Recent research has also demonstrated that small-molecule peptides could help relieve colitis symptoms [8,12,13]. As a small-molecule active peptide, tripeptides can be wholly absorbed by the body and targeted at a specific site to exert good functional properties. Tripeptides have also been reported to prevent and relieve symptoms of colitis. Lys-Pro-Val (KPV) can be transported into the cytosol in the intestine by binding to the oligopeptide transporter (PepT1), further achieving real-time tracking and visualization of the role of intracellular KPV on ulcerative colitis [14]. Meanwhile, Xiao et al. [15] combined KPV with hyaluronic acid (HA) to obtain polymeric nanoparticles (HA-KPV-NPs), which can be targeted to release in the intestines, effectively inhibiting the production of inflammatory factors and alleviating colitis symptoms. Gly-Pro-Ala (GPA) has also been reported to prevent colitis by increasing AMPK phosphorylation and inhibiting the NLRP3 inflammasome [16]. However, no systematic studies have revealed the underlying mechanisms of the 8000 tripeptides in ameliorating colitis symptoms.

Network pharmacology is an interactive network based on the concept of “disease-gene-target-compounds”, which treats the intervention and effect of active compounds on the disease network from a systematic and comprehensive perspective to reveal the complex mechanisms of active compounds [17,18,19]. This study uses a computer-aided screening strategy to explore tripeptides’ intervention mechanism and molecular targets for acute colitis. Furthermore, the interaction mechanisms between hub targets and tripeptides were revealed through molecular docking. The current study identifies which colitis-related targets tripeptides might interact with. Moreover, the present study can also provide precise target information for research on preventing and alleviating colitis using tripeptides.

## 2. Results

### 2.1. Network Interaction Analysis

**Figure 1:**
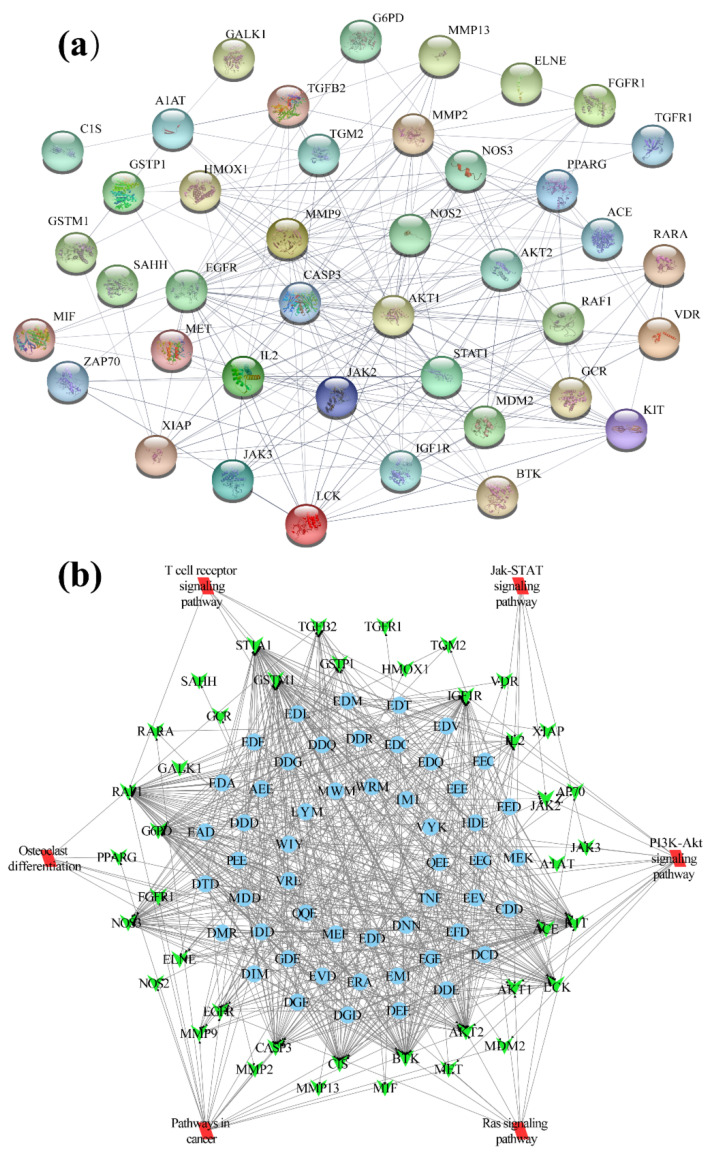
Protein-protein interaction network (**a**) and tripeptides-targets-pathway interaction network diagram (**b**). The green nodes in (**b**) represent the hub targets, the blue nodes represent the tripeptides, and the red nodes stand for the significant signaling pathways.

Based on the pharmacology analysis results, 41 predicted gene targets were screened to construct the set hub targets (Table 1). The PPI network among the target set’s proteins was first analyzed using the STRING database. Figure 1a shows a network with 41 nodes and 225 edges. The results of the topological network analysis showed hub targets with a degree value ≥ 15 played a vital role in the network (Table 2), including AKT1, EGFR, MMP9, CASP3, MMP2, IL2, STAT1, and JAK2.

A tripeptide-target network performed the complex interaction between tripeptides and the screened hub targets. Based on the PharmMapper and GeneCards analysis, tripeptides (related to the most hub targets) were summarized and ranked in the present study. Fifty-two tripeptides were chosen for further tripeptide-target PPI analysis. In Figure 1b, the green nodes represent the hub targets, while the blue nodes denote the 52 tripeptides. The topological analysis of tripeptides and hub targets was also determined (Table S2). Targets GSTM1, BTK, RAF1, G6PD, KIT, ST1A1, C1S, IGF1R, NOS3, AKT2, LCK, CASP3, and ACE showed a high degree value (>28).

### 2.2. Hub Targets GO Biological Functions Enrichments

**Figure 2:**
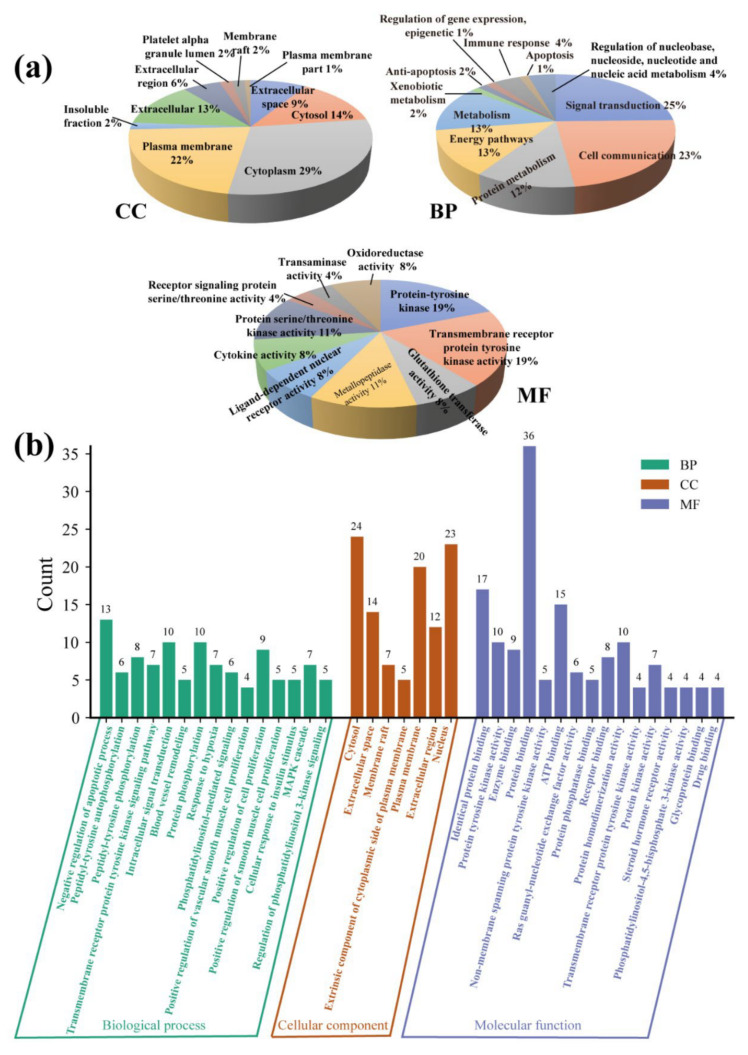
Hub targets GO (gene ontology) enrichment. (**a**) Analysis by the FunRich software (Ver. 3.1.3); (**b**) analysis by the DAVID database (Ver. 6.8) with a *p*-value < 0.001.

GO functional enrichment was performed based on both FunRich (Ver. 3.1.3) and DAVID software (Ver. 6.8) to investigate the function of the selected hub target genes. Figure 2a shows the top ten GO terms based on the analysis of FunRich. Among them, hub targets were enriched in the following cellular component (CC) terms: cytoplasm (30%), plasma membrane, and cytosol (14%). In addition, the hub targets were mainly located in the biological process (BP) pathway terms: signal transduction (25%), cell communication (23%), energy (13%), and metabolism (13%) pathways. For molecular function (MF), the selected hub targets were enriched in terms of protein-tyrosine kinase activity (19%), transmembrane receptor protein tyrosine kinase activity (19%), metallopeptidase activity (19%), metallopeptidase activity (11%), and protein serine/threonine kinase activity (11%).

Furthermore, the DAVID database’s GO enrichment of significant terms for CC, BP, and MF were ranked by *p*-values and are exhibited as bar charts in Figure 2b. The top three highly enriched BP terms contained the negative regulation of the apoptotic process (13 genes, *p*-value = 3.81 × 10^−10^), intracellular signal transduction (10 genes, *p*-value = 3.42 × 10^−7^), and the protein phosphorylation term (10 genes, *p*-value = 9.60 × 10^−7^). The top three CC terms were the cytosol (24 genes, *p*-value = 3.13 × 10^−8^), nucleus (23 genes, *p*-value = 7.30 × 10^−4^), and plasma membrane (20 genes, *p*-value = 4.56 × 10^−4^), respectively. Meanwhile, the top three enrichment MF categories were protein binding (36 genes, *p*-value = 2.25 × 10^−6^), ATP binding (15 genes, *p*-value = 4.39 × 10^−6^), and protein homodimerization activity (10 genes, *p*-value = 4.12 × 10^−5^).

### 2.3. Hub Targets KEGG Pathway Enrichments

**Figure 3:**
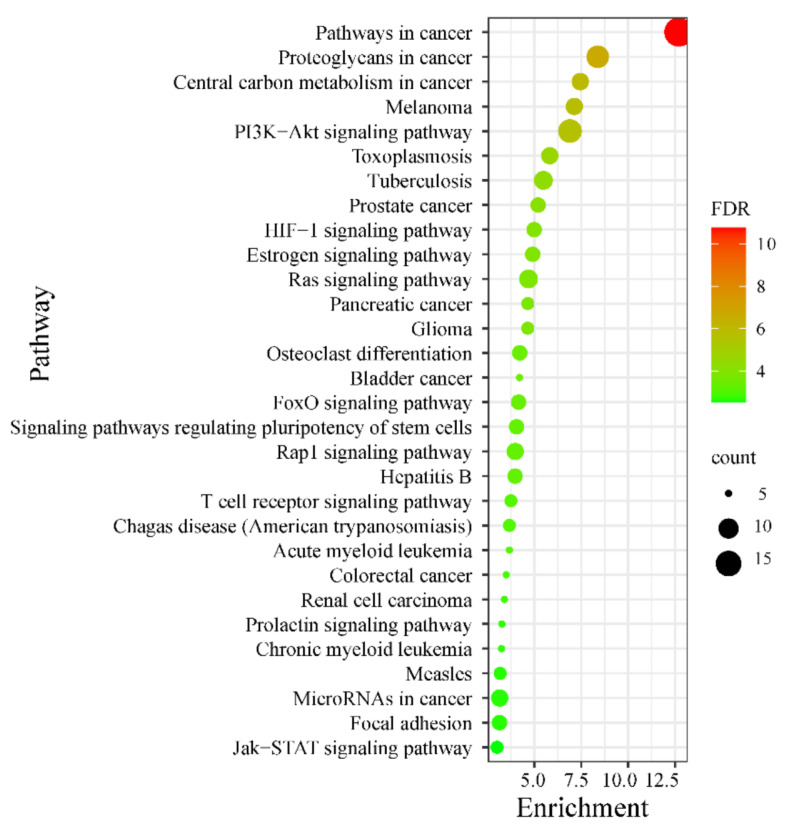
Hub targets KEGG (Kyoto Encyclopedia of Genes and Genomes) pathway enrichment, with a *p*-value < 0.001. The point sizes were positive with the gene numbers in the enrichment pathways, and the color of the point corresponds to the FDR ranges.

The top 30 significant KEGG signaling pathways that the predicted hub targets participated in (*p*-value < 0.001) were further investigated and are shown in a bubble map (Figure 3). These 30 involved signaling pathways were mainly enriched in the pathways related to inflammatory, immunomodulatory, and cell proliferation and differentiation (Table S3). These included the HIF-1 signaling pathway (including targets NOS2, NOS3, AKT2, HMOX1, AKT1, EGFR, and IGF1R), the Ras signaling pathway (including targets ZAP70, AKT2, KIT, AKT1, RAF1, MET, EGFR, FGFR1, and IGF1R), the T cell receptor signaling pathway (including targets ZAP70, LCK, AKT2, AKT1, RAF1, and IL2), the Jak-STAT signaling pathway (containing targets STAT1, AKT2, AKT1, JAK2, JAK3, and IL2), and the PI3K-Akt signaling pathway (including targets NOS3, EGFR, IL2, IGF1R, AKT2, KIT, MDM2, AKT1, JAK2, RAF1, JAK3, MET, and FGFR1). Moreover, the hub targets in both the PI3K-Akt and Ras signaling pathways were mapped to those two pathways (in pink symbols) (Figure S1). Furthermore, the significant signaling pathways were also considered to build the tripeptides-target-pathway network in Figure 1b. This complex network diagram makes it easy to find the interaction between those signaling pathways. Thus, tripeptides may intervene in acute colitis by regulating multiple signaling pathways.

### 2.4. Expression of Hub Targets in Organs Site

**Figure 4:**
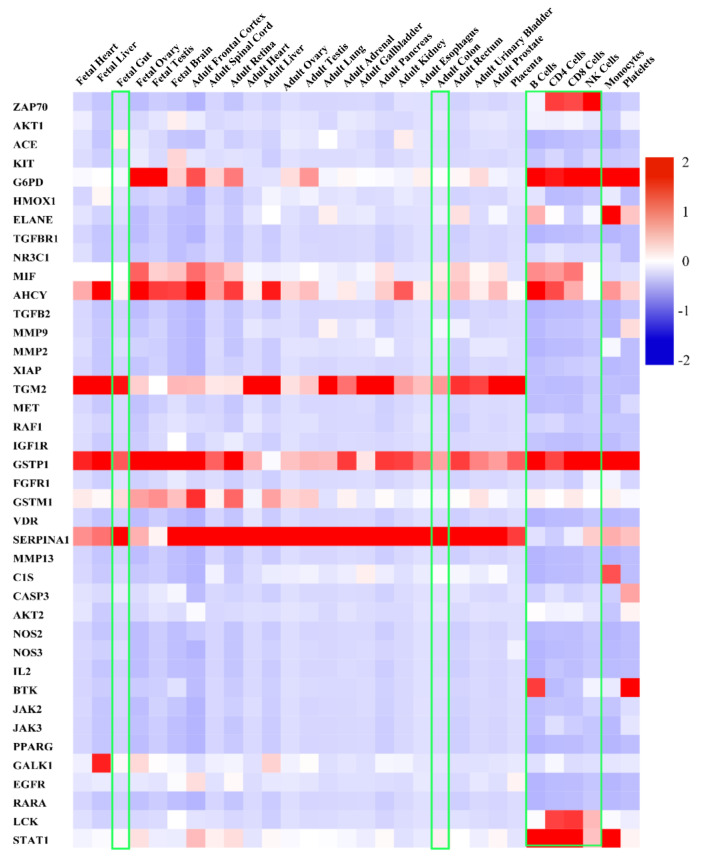
Analysis of the expression of the selected hub targets in the organs based on FunRich software (Ver. 3.1.3). Horizontal coordinates represent organs, while the ordinate coordinates express the hub targets. And the red color represents the high expression of the hub targets in organs, and blue represents the low expression. The green box represents the hub targets involved in the key organs which related to colitis.

The FunRich software (Ver. 3.1.3) revealed the selected 41 hub targets’ organ expression sites (Figure 4). The above 41 hub targets can be expressed in multiple organs and tissues of the human body. Among them, the above targets were significantly related to the organs of the fetal gut, adult colon, and inflammation regulation-connected cells (B cells, CD4 cells, CD8 cells, and NK cells). Hub gene targets (ZAP70, G6PD, MIF, AHCY, LCK, and STAT1) were significantly positively correlated with B cells, CD4 cells, CD8 cells, and NK cells, which showed the opposite trend in both fetal gut and adult colon. Hub gene targets (ACE, KIT, HMOX1, TGFB2, MMP9, MMP2, XIAP, TGM2, MET, RAF1, IGF1R, VDR, MMP13, CIS, CASP3, NOS2, NOS3, IL2, JAK2, JAK3, PARG, EGFR, and RARA) were significantly negatively related to the above inflammation regulation-associated cells. According to the above result, it is reasonable to use the selected hub target as a potential target to regulate acute colitis.

### 2.5. Molecular Docking Results of Tripeptide and Hub Target

Topological analysis results of the thirty hub targets showed that targets of AKT1, EGFR, and MMP9 had a higher “degree value” and “closeness centrality”, as shown in Table 2; thus, targets AKT1, EGFR, and MMP9 were chosen for molecular docking to further verify the correctness of the above analysis.

Reference ligands obtained from AKT1, EGFR, and MMP9 crystal structures, were first considered to optimize the rationality docking conditions. The above three reference ligand structures had highly overlapped before and after docking with RMSD (root-mean-square deviation) values of 0.83 Å, 1.46 Å, and 3.36 Å, respectively (Figure S2). Meanwhile, the binding energy of the reference ligand to AKT1, EGFR, and MMP9 were −14.30, −14.54, and −9.47 kcal/mol, respectively (Table 3), which means the optimized docking conditions were reasonable for subsequent tripeptide-target docking.

After optimizing the docking conditions, the selected 52 tripeptides were all docked with the above three targets’ protein crystal structures, respectively. Results revealed that these 52 tripeptides showed lower binding energy with the targets of AKT1, EGFR, and MMP9 (all binding energy was less than −6.67 kcal/mol, Table 3). The binding energy of the tripeptides (DDD, DDE, EDD, GDE, EEE, CDD, EED, and EDT) with the target AKT1 were all lower than the reference ligand-1, which ranged from −14.39 to −15.86 kcal/mol (Table 3). The binding energy of the 52 tripeptides with the target EGFR was between −7.53 and −13.60 kcal/mol, especially for the tripeptides EDD, EEE, and IDD. The top three tripeptides with lower binding energy (ranging from −12.31 to −11.63 kcal/mol) with the target MMP9 were tripeptides MEK, VYK, and WIY, respectively. 

Moreover, based on the lower binding energy in Table 3, the docking interaction details of the top three tripeptides with hub targets AKT1, EGFR, and MMP9 were further analyzed using Ligplot software (Ver. 2.2), respectively. In Figure S3, tripeptide-target complexes’ binding sites were mainly centralized on the hydrophobic depressions of target crystal structures, and the binding site’s details are shown in Table S4.

**Figure 5:**
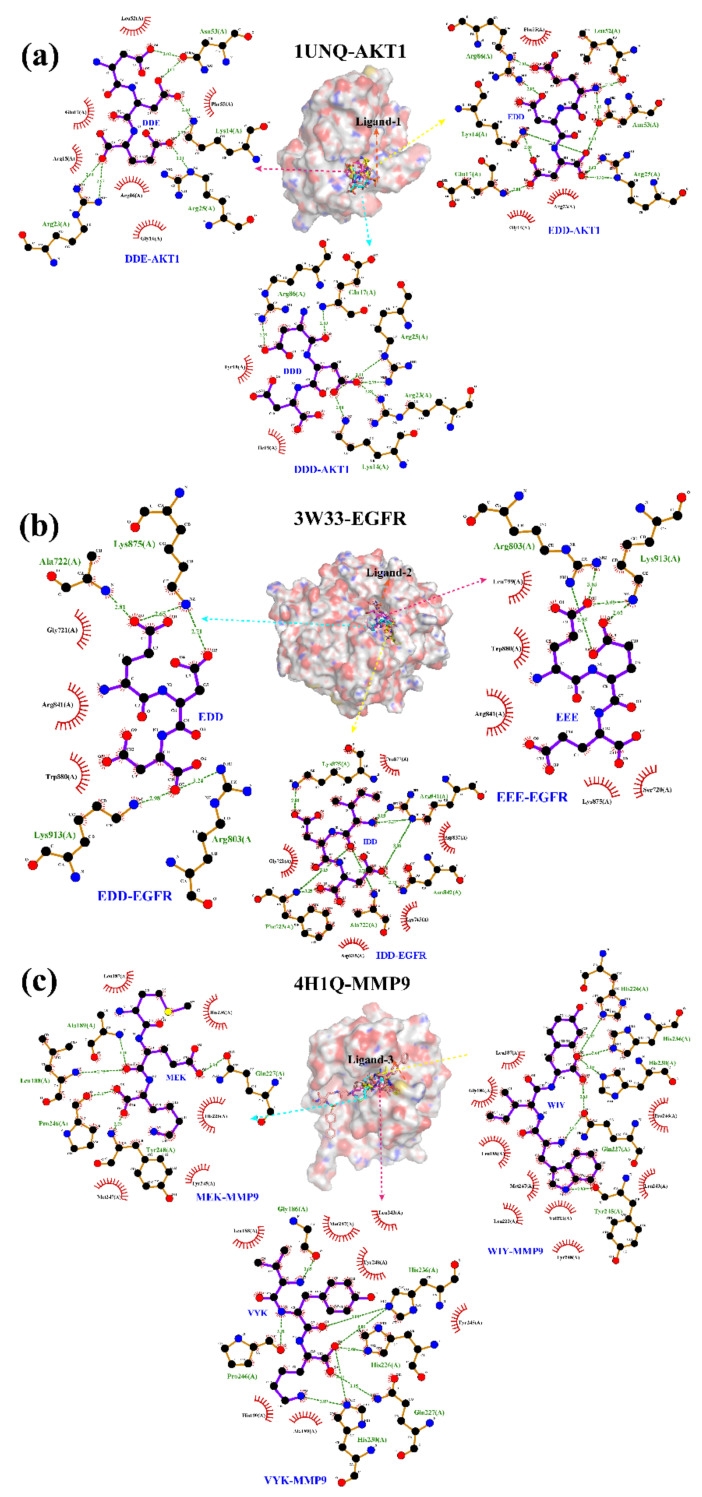
Tripeptides docked into the binding site of the selected target (AKT1 (**a**), EGFR (**b**), and MMP9 (**c**)) crystal structures using the Ligplot software (Ver. 2.2). The peptides are presented as a purple stick model. Hydrogen bonds are indicated by dashed lines between the atoms involved, while an arc represents hydrophobic contacts with spokes radiating towards the ligand atoms they contact.

In Figure 5a, residues (Lys14, Glu17, Arg23, Arg25, and Arg86) of AKT1 generated hydrogen bond interactions with DDD, with distances of 2.80 Å, 2.83 Å, 2.88 Å, 3.11 Å, and 2.75 Å, respectively, are shown. The hydrogen-bonding interaction details of DDE-AKT1 contained the amino acid residues Lys14 (2.60 Å, 2.77 Å), ARG23 (2.61 Å, 2.97 Å), ARG25 (3.21 Å), Asn53 (3.073 Å). In addition, the residues Arg15, Gly16, Glu17, Leu52, Phe55, and Arg86 of AKT1 generated hydrophobic interactions with tripeptide DDE. Meanwhile, EDD tripeptides also revealed hydrogen bond interactions with the amino acid residues Lys14 (2.68 Å, 2.91 Å), Glu17 (2.80 Å), Arg25 (2.62 Å, 3.32 Å), Leu52 (2.74 Å), Asn53 (2.45 Å, 2.83 Å), and Arg86 (2.61 Å, 2.95 Å) of AKT1 (Figure 5a). The above result indicated the critical amino acid residues of AKT1 were Lys14, Glu17, Arg25, and Arg86. Figure 5b shows the bonding details of tripeptides with target EGFR. Residues Ala722, Arg803, Lys875, and Lys913 of EGFR generated conventional hydrogen bond interactions with EDD, with distances of 2.81 Å, 3.24 Å, 2.65 Å, and 2.98 Å, respectively. Meanwhile, EDD also hydrophobically interacted with the EGFR residues Gly721, Arg841, and Trp880. Tripeptide EEE also revealed a hydrogen bond interaction with the residues Arg803 (3.03 Å, 2.95 Å) and Lys913 (3.09 Å, 2.65 Å) of EGFR, and showed hydrophobia with the residues Ser720, Leu799, Arg841, Trp880, and Lys875 of EGFR. IDD generated hydrogen bond interactions with the residues Ala722 (2.78 Å), Phe723 (3.25 Å, 3.15 Å), Arg841 (3.13 Å, 3.27 Å, 3.06 Å), Aan842 (2.73 Å), and Lys875 (2.84 Å) of EGFR. In addition, IDD displayed hydrophobic interactions with the residues Gly721, Lys745, Asp837, Asp855, and Pro877 of EGFR. Based on the above result, the critical amino acid residues of EGFR were Ala722, Arg803, Arg841, Lys875, and Lys913. The bonding details of MMP9 tripeptides were also analyzed (Figure 5c). The residues Leu188, Ala189, Gln227, Pro246, and Tyr248 of MMP9 generated hydrogen bond interactions with MEK. MEK also displayed hydrophobic interactions with MMP9′s amino acid residues Leu187, His226, His236, Tyr245, and Met247. Meanwhile, the residues Gly186 (2.65 Å), His226 (2.68 Å), Gln227 (3.15 Å), His230 (3.75 Å, 2.89 Å), His236 (3.09 Å, 3.08 Å), and Pro246 (3.31 Å) of MMP9 demonstrated hydrogen-bonding interactions with VYK. Further, VYK also displayed hydrophobic interactions with the residues Leu188, Ala189, His190, Leu243, Tyr245, Met247, and Tyr248 of MMP9. The residues His226 (2.89 Å), Gln227 (2.83 Å, 2.81 Å), His230 (3.10 Å), His236 (2.64 Å), and Tyr245 (2.93 Å) of MMP9 also generated hydrogen bond interactions with WIY. Thus, the critical amino acid residues of MMP9 were Leu188, Ala189, His226, Gln227, His236, Tyr245, Pro246, and Tyr248.

## 3. Discussion

Acute colitis is a medical emergency and requires prompt recognition, evaluation, and intervention [20]. During the process of colitis, inflammation impairs the colonic mucosal barrier and increases the permeability of the epithelial barrier, further inducing bacterial invasion into underlying tissues and activating the regulation of the body’s immune system [15,21]. Thus, active ingredients with anti-oxidant and anti-inflammatory activities are expected to relieve colitis symptoms.

As an alternative nutritional material, tripeptides have also been reported to have the ability to be anti-oxidants, anti-inflammatory, and alleviate colitis [14,15,16]. Based on the PharmMapper and Genecards databases, 8000 food-derived tripeptides were used as research objects in the present study to explore potential intervention targets for colitis. A total of 41 acute colitis-related hub targets mainly interacting with tripeptides were screened out. The target-target interaction network was further constructed with 41 nodes and 225 edges. Targets AKT1 (RAC-alpha serine/threonine-protein kinase), EGFR (epidermal growth factor receptor), and MMP9 (matrix metalloproteinase 9) were identified as the top three hub targets with a “degree value” > 23 and “closeness centrality” > 0.70.

Bioinformatic analyses focused on the GO and KEGG pathway enrichment of tripeptides intervention with acute colitis-related targets. The GO results showed that the 41 hub targets were mainly enriched in terms of the cytoplasm, plasma membrane, cytosol, signal transduction, cell communication, protein-tyrosine kinase activity, transmembrane receptor protein tyrosine kinase activity, and metallopeptidase activity. KEGG enrichments mainly contained HIF-1, Ras-, T cell receptors, Jak-STAT, and PI3K-Akt signaling pathways. Among them, the PI3K-Akt signaling pathway has been reported to accelerate the progression of ulcerative colitis by activating the inflammatory signaling pathway [22]. Lee et al. found that PI3K-Akt signaling can interfere with cell survival by regulating the expression of epithelial cell receptors [23].

Moreover, Ras is highly expressed in colitis and can also be used as a therapeutic target in inflammatory diseases [24]. Herein, the selected hub targets mainly participated in the PI3K-Akt and Ras-signaling pathways, especially for the hub targets AKT1 and EGFR. EGFR is a type 1 tyrosine kinase receptor with an extracellular ligand-binding domain and an intracellular portion that contains a tyrosine kinase domain [25]. EGFR has been reported to participate in many biological functional processes, such as the proliferation, differentiation, and survival of cells; meanwhile, activating EGFR can help to alleviate inflammation and further limit the progression of colitis lesions [26,27,28]. Matrix metalloproteinases (MMPs) are zinc-dependent neutral endopeptidases with proteolytic activity against extracellular matrix proteins [29]. MMP9 is a unique MMP, which was highly expressed in inflammation, especially in the samples of enteritis and bowel cancer tissues [30,31]. Furthermore, MMP9 can mediate the EGFR-AKT/ERK pathway, further participating in angiogenesis [32].

This paper uses molecular docking to verify the accuracy of the above bioinformatics analysis, and the results confirmed that tripeptides could enter the hydrophobic domain of AKT1, EGFR, and MMP9. Further, the binding mode of the tripeptide-target was mainly hydrogen-bonding and hydrophobic interaction. The above tripeptide-protein binding mode was consistent with the result reported by Kesari et al. [33].

Additionally, the tripeptide-target binding energies mostly ranged from −7.13 to −15.86 kcal/mol, which means tripeptides can bind tightly to the hub targets related to acute colitis. Therefore, we can confirm that tripeptides may alleviate colitis through pleiotropic pathways, including the Ras- and PI3K-Akt signaling pathways.

## 4. Material and Methods

### 4.1. Construction of Tripeptide Set and Prediction of Colitis-Related Targets

Firstly, the structures of 8000 food-derived tripeptides were constructed. Then, the UCSF Chimera software (Ver. 1.15) was used to obtain the tripeptide energy minimization structures [2]. Afterward, the 8000 food-derived tripeptides were uploaded into the PharmMapper database (http://www.lilab-ecust.cn/pharmmapper/index.php; accessed on 8 July 2021) to predict the potential colitis-related targets [34]. Based on the Fit score value (>4.0), the predicted targets were summarized and used the U’niProt database (http://www.uniprot.org/; accessed on 16 November 2021) to exclude the same targets and non-Homo sapiens targets. Meanwhile, targets related to colitis obtained from the GeneCards Human database (https://www.genecards.org; accessed on 18 November 2021) were used to improve the objective tripeptide hub targets set.

### 4.2. Construction of Protein-Protein Interaction (PPI) Network

The PPI network was constructed using the STRING database (http://string-db.org/; accessed on 25 November 2021) to assess the potential interactions between the 41 screened hub targets [35]. The interactions between hub targets or tripeptide-hub targets were then displayed using Cytoscape software (Ver. 3.6.1, https://cytoscape.org/; accessed on 2 December 2021) [36]. Further, the topological characteristics of PPI networks were also analyzed using Cytoscape’s analytical tool [37,38].

### 4.3. Analysis of GO and KEGG Pathway Enrichment

The DAVID database (http://david.abcc.ncifcrf.gov/home.jsp; accessed on 20 December 2021) has been reported to help integrate functional genomic annotations with intuitive graphical summaries [5,39]. The functional enrichment analysis tool (FunRich), is also widely used to analyze the functional enrichment and interaction network of genes/proteins [40,41]. In this study, the hub tripeptide’s potential targets’ GO (gene ontology) and KEGG (Kyoto encyclopedia of genes and genomes) signaling pathways were systematically analyzed using the DAVID (Ver. 6.8) and FunRich databases. During the analysis process, the significant enriched GO terms (*p*-value < 0.05) and KEGG pathways (*p*-value < 0.001) were considered for further evaluation. Finally, the data were visually displayed using the free bioinformatics database (http://www.bioinformatics.com.cn/; accessed on 24 December 2021).

### 4.4. Hub Target’s Expression Organs Site

This study used FunRich software (Ver. 3.1.3) to research the selected hub target’s main organ site expression based on the “Human proteome” database. The result was displayed in the form of a heatmap. The positively correlated high-expressed targets in the organs are shown in red, while the low-expressed targets are marked in blue. The higher the expression level of the target, the darker the color.

### 4.5. Molecular Docking

Subsequently, AutoDock 4.2 was used to evaluate the interaction between tripeptides and the above three hub targets [42]. Based on PPI analysis results (the top three hub targets with higher degree values), the crystal structures of AKT1 (1unq, resolution: 0.98 Å) [43], EGFR (3w33, resolution: 1.7 Å) [44], and MMP9 (4h1q, resolution: 1.59 Å) [45] were chosen as the receptor proteins from the RCSB protein data bank (http://www.rcsb.org/pdb; accessed on 3 January 2022) [46] for further molecular docking. The filtering criteria for selecting reporter proteins are as follows: organism “Homo”, “Resolution < 2.0”, and with a unique ligand. Before docking, the unique ligand and water molecules of AKT1, EGFR, and MMP9 crystal structures were removed. Then, hydrogen atoms were added to the three hub targets, respectively. Tripeptide molecular structures were built as the ligand for docking. Before docking, the energy of all tripeptides was minimized by adding the CHARMM force field [47,48].

To verify the rationality-optimized docking conditions, the reference ligands of AKT1, EGFR, and MMP9 crystal structures were first considered. A Lamarckian genetic algorithm as the search engine searched 200 runs. During docking, the grid spacing was set to 0.375 Å [49]. The final docking condition details of each receptor target after completing docking condition optimization are shown in Table S1. The hydrogen bonds, hydrophilic, and coordination interactions between residues of receptor proteins’ active sites and the tripeptide’s poses were identified using Discovery Studio Visualizer (Ver. 4.1). Finally, Ligplot software (Ver. 2.2) was used to display the details of the interactions between tripeptide-targets [50].

## 5. Conclusions

Based on the computer-aided screening strategy, this study has systematically investigated the potential effects of 8000 tripeptides to alleviate acute colitis, including the possible pleiotropic pathways. Tripeptides play a role in relieving colitis by regulating signaling pathways, such as inflammation, immunomodulation, and cell proliferation and differentiation, especially in the Ras- and PI3K-Akt signaling pathways. This work provides a computer-aided method for screening potential functional targets of active ingredients, which can provide a reference for the functional mining of food-derived peptides.

## Figures and Tables

**Table 1:** List of predicted genes of tripeptides intervene in acute colitis.

No.	Gene	Protein Target	UniProt ID
1	A1AT	Alpha-1-antitrypsin	P01009
2	ACE	Angiotensin-converting enzyme	P12821
3	AKT1	RAC-alpha serine/threonine-protein kinase	P31749
4	AKT2	RAC-beta serine/threonine-protein kinase	P31751
5	BTK	Tyrosine-protein kinase BTK	Q06187
6	C1S	Complement C1s subcomponent	P09871
7	CASP3	Caspase-3	P42574
8	EGFR	Epidermal growth factor receptor	P00533
9	ELNE	Neutrophil elastase	P08246
10	FGFR1	Fibroblast growth factor receptor 1	P11362
11	G6PD	Glucose-6-phosphate 1-dehydrogenase	P11413
12	GALK1	Galactokinase	P51570
13	GCR	Glucocorticoid receptor	P04150
14	GSTM1	Glutathione S-transferase Mu 1	P09488
15	GSTP1	Glutathione S-transferase P	P09211
16	HMOX1	Heme oxygenase 1	P09601
17	IGF1R	Insulin-like growth factor 1 receptor	P08069
18	IL2	Interleukin-2	P60568
19	JAK2	Tyrosine-protein kinase JAK2·	O60674
20	JAK3	Tyrosine-protein kinase JAK3	P52333
21	KIT	Mast/stem cell growth factor receptor Kit	P10721
22	LCK	Interleukin-2	P06239
23	MDM2	E3 ubiquitin-protein ligase Mdm2	Q00987
24	MET	Hepatocyte growth factor receptor	P08581
25	MIF	Macrophage migration inhibitory factor	P14174
26	MMP13	Collagenase 3	P45452
27	MMP2	72 kDa type IV collagenase	P08253
28	MMP9	Matrix metalloproteinase-9	P14780
29	NOS2	Nitric oxide synthase, inducible	P35228
30	NOS3	Nitric oxide synthase, endothelial	P29474
31	PPARG	Peroxisome proliferator-activated receptor gamma	P37231
32	RAF1	RAF proto-oncogene serine/threonine-protein kinase	P04049
33	RARA	Retinoic acid receptor alpha	P10276
34	SAHH	Adenosylhomocysteinase	P23526
35	STAT1	Signal transducer and activator of transcription 1-alpha/beta	P42224
36	TGFB2	Transforming growth factor beta-2 proprotein	P61812
37	TGFR1	TGF-beta receptor type-1	P36897
38	TGM2	Protein-glutamine gamma-glutamyltransferase 2	P21980
39	VDR	Vitamin D3 receptor	P11473
40	XIAP	E3 ubiquitin-protein ligase XIAP	P98170
41	ZAP70	Tyrosine-protein kinase ZAP-70	P43403

**Table 2:** Topology analysis of the hub targets protein-protein interactions.

No.	Name	Betweenness Centrality	Closeness Centrality	Clustering Coefficient	Degree
1	AKT1	0.1690	0.8000	0.3471	30
2	EGFR	0.1477	0.7547	0.3276	29
3	MMP9	0.0949	0.7143	0.4275	24
4	CASP3	0.0423	0.6897	0.4819	24
5	MMP2	0.0854	0.6349	0.4211	20
6	IL2	0.0326	0.6250	0.4795	19
7	STAT1	0.0221	0.6061	0.5294	18
8	JAK2	0.0269	0.6250	0.4902	18
9	PPARG	0.0093	0.5882	0.6286	15
10	HMOX1	0.0354	0.5797	0.5055	14
11	NOS3	0.0094	0.5797	0.6154	14
12	MDM2	0.0139	0.5797	0.5165	14
13	LCK	0.0094	0.5634	0.6264	14
14	KIT	0.0093	0.5634	0.5897	13
15	AKT2	0.0066	0.5797	0.6795	13
16	NOS2	0.0041	0.5479	0.6970	12
17	IGF1R	0.0060	0.5714	0.6970	12
18	XIAP	0.0658	0.5556	0.5636	11
19	GCR	0.0034	0.5405	0.6727	11
20	RAF1	0.0036	0.5556	0.7273	11
21	ACE	0.0023	0.5479	0.8000	10
22	VDR	0.0032	0.5263	0.6111	9
23	TGM2	0.0070	0.5479	0.7143	8
24	TGFB2	0.0073	0.5405	0.7500	8
25	JAK3	0.0007	0.5195	0.8214	8
26	BTK	0.0025	0.5195	0.7500	8
27	RARA	0.0008	0.5128	0.7619	7
28	MMP13	0.0007	0.5195	0.8571	7
29	FGFR1	0.0009	0.5195	0.8095	7
30	ALAT	0.0994	0.5195	0.3810	7
31	MET	0.0004	0.5195	0.9048	7
32	GSTP1	0.0128	0.5263	0.4667	6
33	ZAP70	0.0003	0.4819	0.9000	5
34	MIF	0.0000	0.4938	1.0000	4
35	G6PD	0.0001	0.5000	0.8333	4
36	GSTM1	0.0015	0.4301	0.3333	3
37	TGFR1	0.0000	0.4598	1.0000	2
38	SAHH	0.0000	0.3604	0.0000	1
39	C1S	0.0000	0.3448	0.0000	1
40	ELNE	0.0000	0.3922	0.0000	1
41	GALK1	0.0000	0.3448	0.0000	1

**Table 3:** Binding free energy of ligand-tripeptides (kcal/mol).

Ligands	AKT1	EGFR	MMP9	Ligands	AKT1	EGFR	MMP9
	Binding Energy (kcal/mol)				Binding Energy (kcal/mol)		
Ligand-1	−14.30	-	-	EEC	−13.13	−10.14	−9.27
Ligand-2	-	−14.54	-	EED	−14.39	−12.53	−8.09
Ligand-3	-	-	−9.47	EEE	−14.67	−12.84	−7.77
CDD	−14.60	−11.81	−10.50	EEG	−13.54	−10.05	−7.82
DCD	−13.68	−10.15	−9.93	EEH	−12.97	−7.80	−9.45
DDD	−15.30	−11.43	−8.13	EEV	−13.04	−10.61	−8.26
DDE	−15.86	−11.77	−7.23	EFD	−12.75	−10.85	−11.61
DDG	−13.63	−11.16	−9.26	EGE	−14.03	−11.67	−7.47
DDQ	−13.31	−11.52	−6.67	EMI	−11.62	−9.64	−8.11
DDR	−12.74	−10.25	−11.60	EVD	−14.25	−11.80	−8.83
DDS	−14.35	−12.20	−10.54	GDE	−15.25	−12.09	−7.79
DEE	−13.21	−10.56	−6.35	HDE	−12.24	−11.46	−9.48
DGD	−14.34	−12.07	−9.37	IDD	−13.81	−12.83	−8.86
DGE	−12.72	−11.47	−7.13	IMI	−7.88	−7.57	−8.09
DIM	−10.90	−9.63	−9.12	LYM	−8.09	−8.43	−9.84
DMR	−12.48	−11.43	−9.76	MDD	−11.99	−10.67	−10.36
DNN	−13.33	−11.49	−9.91	MEF	−10.29	−9.66	−9.23
DTD	−14.34	−12.42	−8.84	MEK	−11.28	−10.59	−12.31
EAD	−13.78	−11.17	−9.32	MWM	−8.68	−8.95	−9.44
EDA	−12.78	−10.60	−9.20	PEE	−11.16	−10.09	−6.71
EDC	−14.01	−8.53	−9.29	QEE	−13.54	−11.57	−8.12
EDD	−15.52	−13.60	−8.62	QQE	−13.05	−11.09	−9.47
EDE	−13.95	−12.26	−6.95	TNE	−12.95	−11.56	−9.33
EDL	−12.29	−11.09	−9.00	VRE	−11.61	−11.35	−10.79
EDM	−12.23	−11.25	−9.78	VYK	−7.71	−9.92	−11.63
EDQ	−12.62	−11.01	−8.89	WIY	−11.06	−10.27	−12.30
EDT	−14.38	−11.71	−9.21	WRM	−9.16	−10.00	−10.76
EDV	−12.46	−10.90	−9.51				

## Data Availability

Not applicable.

## Supplementary Materials

The following supporting information can be downloaded at: https://www.mdpi.com/article/10.3390/ijms232113471/s1.
