# Supplementary material for: Computer-Aided Screening and Revealing Action Mechanism of Food-Derived Tripeptides Intervention in Acute Colitis
Source: Int J Mol Sci. 2022 Nov 3;23(21):13471. doi: 10.3390/ijms232113471 (PMC9655126; doi:10.3390/ijms232113471)
Supplement: Supplementary file 1 [file ijms-23-13471-s001.zip › Supplement Figure.pdf]

(a)

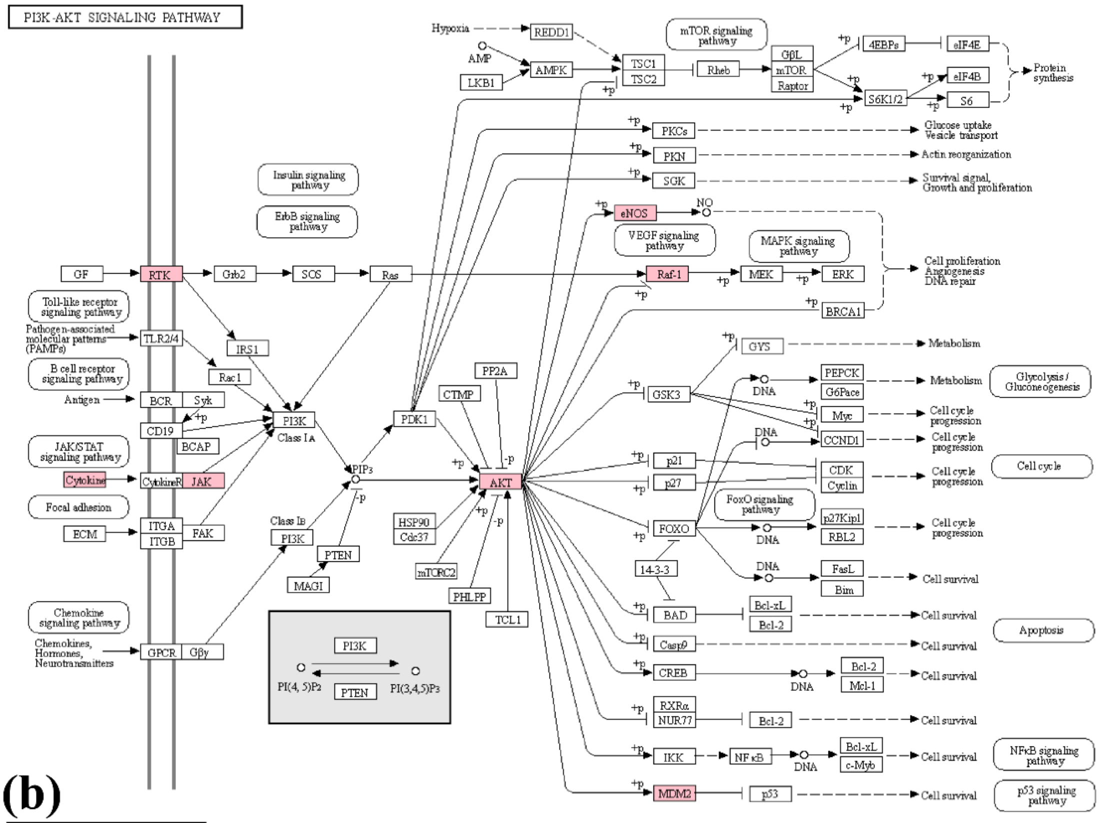

(b)

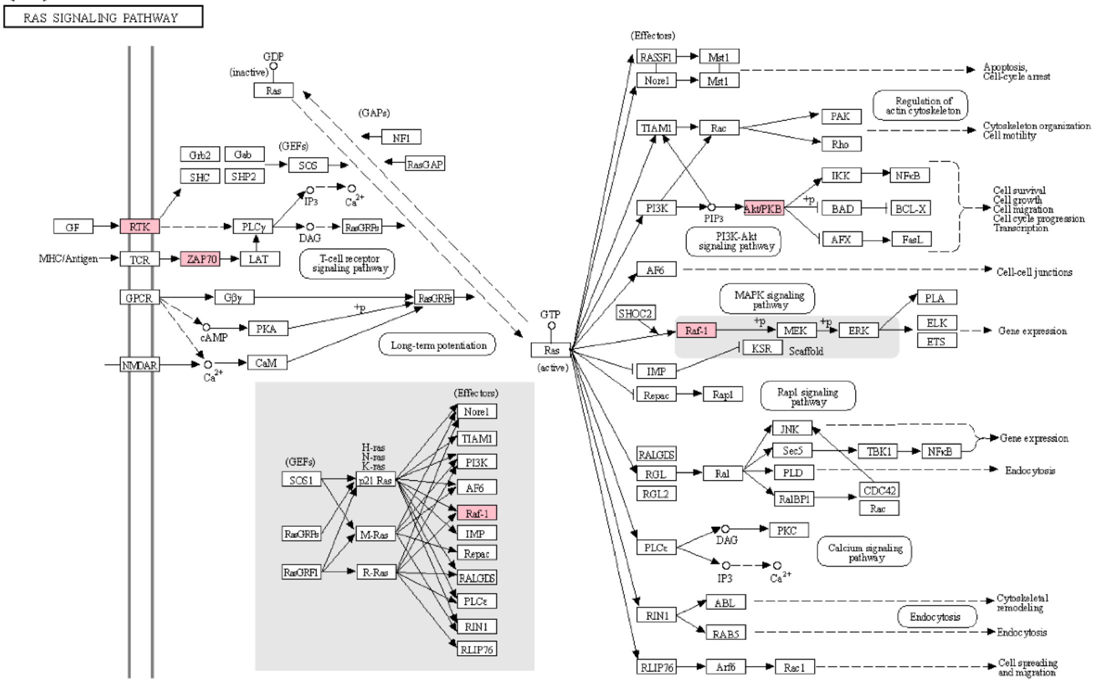

Figure S1 Significant tripeptides' pharmaceutical action genes participate in PI3K-AKT (**a**) and Ras (**b**) signaling pathways. All the matched nodes were colored in red.

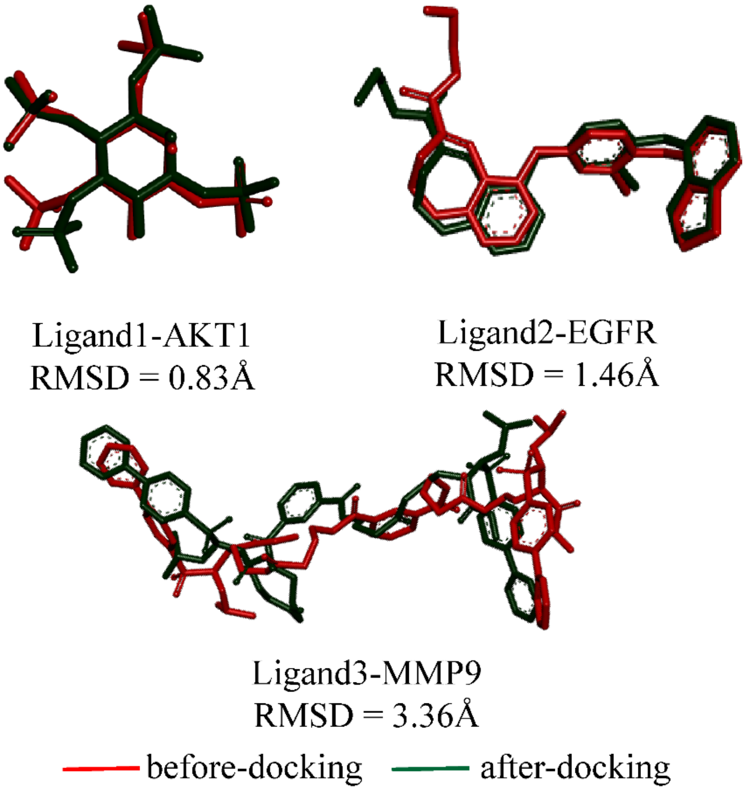

Figure S2 Changeable of the reference ligands structures before and after re-docking

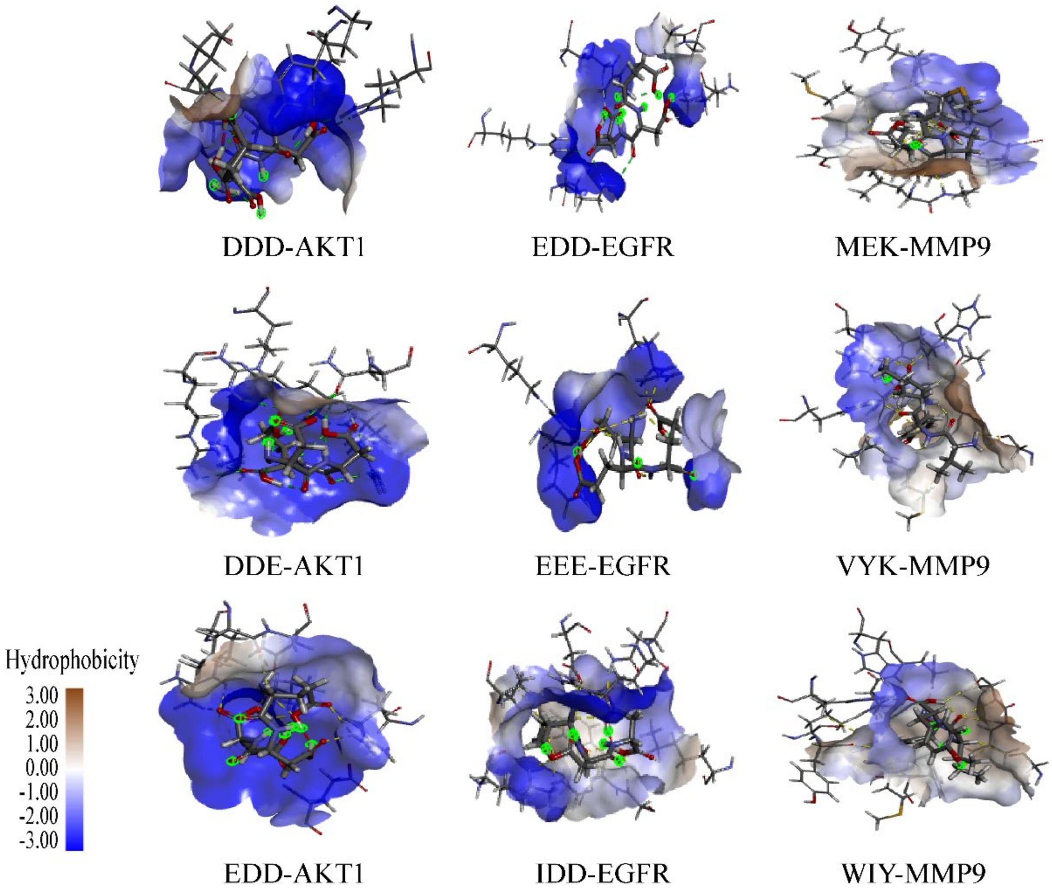

Figure S3 Position of tripeptides in the hydrophobic depression domain of core targets (AKT1, EGFR, and MMP9) crystal structure
