# Supplementary material for: Computer-Aided Screening and Revealing Action Mechanism of Food-Derived Tripeptides Intervention in Acute Colitis
Source: Int J Mol Sci. 2022 Nov 3;23(21):13471. doi: 10.3390/ijms232113471 (PMC9655126; doi:10.3390/ijms232113471)
Supplement: Supplementary file 1 [file ijms-23-13471-s001.zip › Supplement Table.pdf]

Table S1 Setting of Docking conditions

	**x**	**y**	**z**	grid box
AKT1	15.18	24.427	16.345	80 × 80 × 80 Å
EGFR	17.388	32.874	12.434	80 × 80 × 80 Å
MMP9	29.115	5.773	18.728	95 × 90 × 90 Å

Table S2 Topology analysis of the tripeptides - targets interactions (Degree ≥ 15)

Name	Average Shortest Path Length	Betweenness Centrality	Closeness Centrality	Degree	Neighborhood Connectivity	Topological Coefficient
GSTM1	1.5217	0.2039	0.6571	49	11.9796	0.2815
BTK	1.6739	0.0996	0.5974	46	12.6087	0.3317
RAF1	1.8261	0.0573	0.5476	42	13.2381	0.3824
G6PD	1.8478	0.0511	0.5412	41	13.4878	0.3902
KIT	1.8478	0.0606	0.5412	41	13.4634	0.3895
ST1A1	1.8696	0.0503	0.5349	41	13.5610	0.4052
C1S	2.0000	0.0278	0.5000	37	14.2703	0.4576
IGF1R	2.0000	0.0278	0.5000	37	14.2703	0.4576
NOS3	2.0435	0.0258	0.4894	36	14.1944	0.4712
AKT2	2.0652	0.0277	0.4842	35	14.1143	0.4684
LCK	2.1087	0.0316	0.4742	34	14.0882	0.4847
CASP3	2.1957	0.0178	0.4554	33	14.2813	0.5313
ACE	2.1957	0.0296	0.4554	29	13.4138	0.4433
EDC	2.0978	0.0226	0.4767	17	31.5625	0.5993
EDE	2.1630	0.0125	0.4623	16	32.9375	0.6518
EDM	2.0978	0.0307	0.4767	16	32.1250	0.6103
EED	2.0978	0.0142	0.4767	16	33.3125	0.6336
CDD	2.1848	0.0059	0.4577	15	34.9333	0.6925
DDE	2.1848	0.0056	0.4577	15	34.9333	0.6925
DGE	2.1196	0.0093	0.4718	15	34.8667	0.6641
EDL	2.1196	0.0196	0.4718	15	34.4667	0.6562
EEV	2.1196	0.0098	0.4718	15	33.6667	0.6405
IDD	2.1413	0.0300	0.4670	15	33.7333	0.6547
MDD	2.1196	0.0077	0.4718	15	34.9333	0.6654
EDQ	2.1196	0.0121	0.4718	15	33.7333	0.6418
EDD	2.1848	0.0167	0.4577	15	31.4000	0.6204
EGFR	2.6087	0.0073	0.3833	15	13.9333	0.5623
IL2	2.5217	0.0360	0.3966	15	11.9333	0.4049
TGFB2	2.5435	0.0357	0.3932	15	11.7333	0.4128

Table S3 KEGG signaling pathways of the predicted core targets (organism: Mus musculus) (*p-value* < 0.001)

Rank	Term ID	Pathway	Gene count	*p-value*	Matching proteins
1	hsa05200	Pathways in cancer	19	1.86E-13	TGFB2, NOS2, STAT1, GSTP1, MMP2, XIAP, MMP9, EGFR, IGF1R, CASP3, AKT2, KIT, MDM2, RARA, AKT1, PPARG, RAF1, MET, FGFR1
2	hsa05205	Proteoglycans in cancer	12	4.21E-09	TGFB2, AKT2, CASP3, MMP2, MDM2, AKT1, RAF1, MET, MMP9, EGFR, FGFR1, IGF1R
3	hsa05230	Central carbon metabolism in cancer	8	3.56E-08	G6PD, AKT2, KIT, AKT1, RAF1, MET, EGFR, FGFR1
4	hsa05218	Melanoma	8	7.43E-08	AKT2, MDM2, AKT1, RAF1, MET, EGFR, FGFR1, IGF1R
5	hsa04151	PI3K-Akt signaling pathway	13	1.25E-07	NOS3, EGFR, IL2, IGF1R, AKT2, KIT, MDM2, AKT1, JAK2, RAF1, JAK3, MET, FGFR1
6	hsa05145	Toxoplasmosis	8	1.53E-06	TGFB2, NOS2, STAT1, AKT2, CASP3, XIAP, AKT1, JAK2
7	hsa05152	Tuberculosis	9	3.35E-06	TGFB2, NOS2, STAT1, VDR, AKT2, CASP3, AKT1, JAK2, RAF1
8	hsa05215	Prostate cancer	7	6.24E-06	AKT2, MDM2, AKT1, RAF1, EGFR, FGFR1, IGF1R
9	hsa04066	HIF-1 signaling pathway	7	1.03E-05	NOS2, NOS3, AKT2, HMOX1, AKT1, EGFR, IGF1R
10	hsa04915	Estrogen signaling pathway	7	1.24E-05	NOS3, AKT2, MMP2, AKT1, RAF1, MMP9, EGFR
11	hsa04014	Ras signaling pathway	9	2.03E-05	ZAP70, AKT2, KIT, AKT1, RAF1, MET, EGFR, FGFR1, IGF1R
12	hsa05214	Glioma	6	2.22E-05	AKT2, MDM2, AKT1, RAF1, EGFR, IGF1R
13	hsa05212	Pancreatic cancer	6	2.22E-05	TGFB2, STAT1, AKT2, AKT1, RAF1, EGFR
14	hsa04380	Osteoclast differentiation	7	6.09E-05	TGFB2, STAT1, LCK, AKT2, BTK, AKT1, PPARG
15	hsa05219	Bladder cancer	5	6.22E-05	MMP2, MDM2, RAF1, MMP9, EGFR
16	hsa04068	FoxO signaling pathway	7	6.91E-05	TGFB2, AKT2, MDM2, AKT1, RAF1, EGFR, IGF1R

Rank	Term ID	Pathway	Gene count	*p-value*	Matching proteins
17	hsa04550	Signaling pathways regulating pluripotency of stem cells	7	8.83E-05	AKT2, AKT1, JAK2, RAF1, JAK3, FGFR1, IGF1R
18	hsa04015	Rap1 signaling pathway	8	1.06E-04	AKT2, KIT, AKT1, RAF1, MET, EGFR, FGFR1, IGF1R
19	hsa05161	Hepatitis B	7	1.07E-04	TGFB2, STAT1, AKT2, CASP3, AKT1, RAF1, MMP9
20	hsa04660	T cell receptor signaling pathway	6	1.77E-04	ZAP70, LCK, AKT2, AKT1, RAF1, IL2
21	hsa05142	Chagas disease (American trypanosomiasis)	6	2.13E-04	TGFB2, ACE, NOS2, AKT2, AKT1, IL2
22	hsa05221	Acute myeloid leukemia	5	2.13E-04	AKT2, KIT, RARA, AKT1, RAF1
23	hsa05210	Colorectal cancer	5	3.16E-04	TGFB2, AKT2, CASP3, AKT1, RAF1
24	hsa05211	Renal cell carcinoma	5	4.02E-04	TGFB2, AKT2, AKT1, RAF1, MET
25	hsa04917	Prolactin signaling pathway	5	5.32E-04	STAT1, AKT2, AKT1, JAK2, RAF1
26	hsa05220	Chronic myeloid leukemia	5	5.61E-04	TGFB2, AKT2, MDM2, AKT1, RAF1
27	hsa05162	Measles	6	6.64E-04	STAT1, AKT2, AKT1, JAK2, JAK3, IL2
28	hsa05206	MicroRNAs in cancer	8	7.01E-04	TGFB2, CASP3, MDM2, HMOX1, RAF1, MET, MMP9, EGFR
29	hsa04510	Focal adhesion	7	7.17E-04	AKT2, XIAP, AKT1, RAF1, MET, EGFR, IGF1R
30	hsa04630	Jak-STAT signaling pathway	6	9.83E-04	STAT1, AKT2, AKT1, JAK2, JAK3, IL2

Table S4 Results of molecular docking between core targets and peptides

Peptides-target	Hydrogen Bonds	Electrostatic	Hydrophobic
DDD - AKT1	LYS14:HZ3 - DDD:O5; GLU17:HN - DDD:O; ARG25:HE - DDD:O5; ARG25:HH21 - DDD:O3; ARG86:HH21 - DDD:O2; ARG23:HD1 - DDD:O3	-	-
DDE - AKT1	LYS14:HZ1 - DDE:O2; LYS14:HZ3 - DDE:O7; ARG23:HH11 - DDE:O6; ARG23:HH21 - DDE:O6; ARG25:HE - DDE:O7; ARG25:HH21 - DDE:O7; DDE:H4 - ASN53:OD1; DDE:H16 - ASN53:OD1; LYS14:HE1 - DDE:O2	-	ARG86:HE - DDE:O2; ARG86:HH21 - DDE:O3; GLY16:HA1 - DDE:O6; GLY16:HA2 - DDE:O6-
EDD - AKT1	LYS14:HZ1 - EDD:O9; LYS14:HZ2 - EDD:O9; GLU17:HN - EDD:O8; ARG25:HE - EDD:O7; ARG25:HH21 - EDD:O7; ARG86:HE - EDD:O2; ARG86:HH21 - EDD:O2; ARG86:HH21 - EDD:O5; EDD:H - LEU52:O; EDD:H - ASN53:OD1; EDD:H3 - ASN53:OD1; EDD:H10 - LEU52:O	-	-
EDD - EGFR	ALA722:HN - EDD:O2; ARG803:HH22 - EDD:O7; LYS875:HZ1 - EDD:O5; LYS875:HZ2 - EDD:O2; LYS913:HZ1 - EDD:O3; LYS913:HZ3 - EDD:O7	-	ARG841:HD1 - EDD:O9; GLY721:HA1 - EDD:O2
EEE - EGFR	ARG803:HH12 - EEE:O1; LYS913:HZ2 - EEE:O4	-	ARG841:HD1 - EEE:O8; ARG841:HD2 - EEE:O8
IDD - EGFR	ALA722:HN - IDD:O; PHE723:HN - IDD:O; GLY724:HN - IDD:O7; ARG841:HE - IDD:O2; ASN842:HD21 - IDD:O2; LYS875:HZ1 - IDD:O3; LYS875:HE2 - IDD:O3	-	LYS745:HE1 - IDD:O6; IDD:H9 - ASP837:OD2; IDD:H6 - ASP837:OD2; ALA722 - IDD:C5; IDD:C5 - PRO877; PHE723 - IDD:C5

Peptides-target	Hydrogen Bonds	Electrostatic	Hydrophobic
MEK - MMP9	LEU188:HN - MEK:O1; ALA189:HN - MEK:O1; TYR248:HN - MEK:O4; MEK:H3 - PRO246:O; MEK:H14 - GLN227:OE1; MEK:H25 - TYR245:O	-	MET247:HA - MEK:O4; VAL223 - MEK; MEK - LEU188; HIS226 - MEK
VYK - MMP9	VYK:H12 - PRO246:O; VYK:H - GLY186:O; VYK:H4 - GLY186:O; HIS226:HE2 - VYK:O1; GLN227:HE22 - VYK:O4; HIS230:HE2 - VYK:O3; HIS236:HE2 - VYK:O1; VYK:H18 - ALA189:O; HIS190:HA - VYK:O4; HIS230:HD2 - VYK:O4; PRO246:HA - VYK:O1	-	HIS226 - VYK; VYK:C2 - MET247; HIS236 - VYK
WIY - MMP9	LEU188:HN - WIY:O; ALA189:HN - WIY:O; HIS226:HE2 - WIY:O2; HIS230:HE2 - WIY:O2; HIS236:HE2 - WIY:O2; WIY:H - GLN227:OE1; WIY:H3 - TYR245:O; WIY:H27 - ALA189:O; WIY:H8 - GLY186:O	HIS226:NE2 - WIY	HIS226 - WIY; MET247:C,O;TYR248:N - WIY; WIY:C15 - LEU188; WIY - LEU243
